# malERA: An updated research agenda for characterising the reservoir and measuring transmission in malaria elimination and eradication

**DOI:** 10.1371/journal.pmed.1002452

**Published:** 2017-11-30

**Authors:** The malERA Refresh Consultative Panel on Characterising the Reservoir and Measuring Transmission

## Abstract

This paper summarises key advances in defining the infectious reservoir for malaria and the measurement of transmission for research and programmatic use since the Malaria Eradication Research Agenda (malERA) publication in 2011. Rapid and effective progress towards elimination requires an improved understanding of the sources of transmission as well as those at risk of infection. Characterising the transmission reservoir in different settings will enable the most appropriate choice, delivery, and evaluation of interventions. Since 2011, progress has been made in a number of areas. The extent of submicroscopic and asymptomatic infections is better understood, as are the biological parameters governing transmission of sexual stage parasites. Limitations of existing transmission measures have been documented, and proof-of-concept has been established for new innovative serological and molecular methods to better characterise transmission. Finally, there now exists a concerted effort towards the use of ensemble datasets across the spectrum of metrics, from passive and active sources, to develop more accurate risk maps of transmission. These can be used to better target interventions and effectively monitor progress toward elimination. The success of interventions depends not only on the level of endemicity but also on how rapidly or recently an area has undergone changes in transmission. Improved understanding of the biology of mosquito–human and human–mosquito transmission is needed particularly in low-endemic settings, where heterogeneity of infection is pronounced and local vector ecology is variable. New and improved measures of transmission need to be operationally feasible for the malaria programmes. Outputs from these research priorities should allow the development of a set of approaches (applicable to both research and control programmes) that address the unique challenges of measuring and monitoring transmission in near-elimination settings and defining the absence of transmission.

## Abstract 2

Christopher Drakeley and colleagues propose an updated research agenda for characterizing the reservoir and measuring transmission in malaria elimination and eradication.

**Summary points**

- Understanding the sources of transmission (the infectious reservoir) and those at risk of infection at the population level in order to inform programmatic decision-making can progress malaria elimination.
- There is considerable evidence for malaria infections at densities beneath the limit of conventional diagnostics. However, the contribution of these low-density infections to malaria transmission in different settings is not known.
- Characterising the spatial and temporal heterogeneity of the infectious reservoir becomes increasingly important as transmission declines if interventions are to be efficiently implemented to accelerate malaria elimination.
- The proportional contributions of low-density, asymptomatic, and symptomatic infections will differ by malaria typology and will determine the programmatic approach required to reduce transmission.
- *Plasmodium vivax* hypnozoites are undetectable with currently available diagnostics, representing a major barrier to both understanding the transmission reservoir for this parasite and its elimination.
- There is a need to standardise both existing transmission metrics and new metrics with greater sensitivity, particularly for their use in low-transmission settings.

## Introduction

Transmission of malaria requires sexual-stage parasites, gametocytes, in humans to be taken up by female *Anopheles* mosquitoes when they feed. After a period of parasite development, mosquitoes can then infect humans. A break in this cycle at any point interrupts malaria transmission. Malaria control has historically focussed on the reduction of morbidity and mortality of the human host rather than on the interruption of transmission from human to mosquito. Understanding the variation in the relationship between infection (the presence of parasites in an individual or mosquito) and infectiousness (the ability to transmit parasites to a mosquito or human) at different transmission intensities and with different levels of intervention coverage is increasingly recognised as critical in the pursuit of malaria elimination.

In 2011, one of the main conclusions of the Malaria Eradication Research Agenda (malERA) process was the need to develop tools to measure transmission at low levels in elimination contexts. This article summarizes progress made since 2011 and for the first time develops a research agenda addressing the reservoir of transmissible parasites and measuring transmission [1,2]. Findings and recommendations presented here result from a systematic search of the literature and the deliberations of the 2015 malERA Refresh Consultative Panel on characterising the reservoir and measuring transmission, including specialists from field and implementation science, entomology, epidemiology, and basic science.

**Fig 1:**
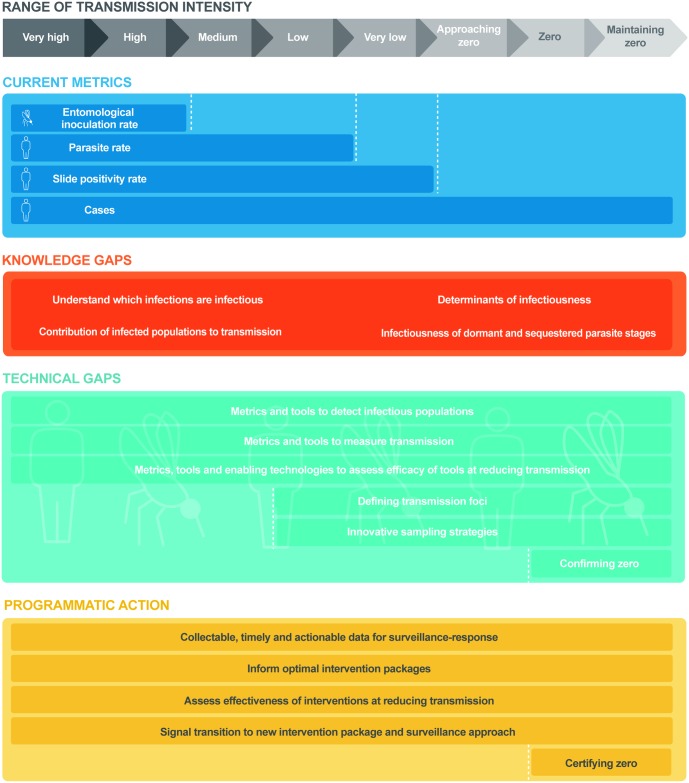
Research needs and programmatic applications in measuring malaria transmission across the transmission spectrum. **Range of malaria transmission intensity** (grey line) from very high intensity to postelimination settings. **Current metrics** (navy blue line) used for routine measurement of malaria transmission at each level of transmission intensity. **Knowledge gaps** (orange line) in understanding the biology and epidemiology of malaria transmission and the infectious reservoir at all levels of transmission intensity. **Technical gaps** (light blue line) in the accurate measurement of transmission at each level of transmission intensity. **Programmatic actions** (yellow line) required for the interruption of transmission and the prevention of reintroduction at each level of transmission intensity.

Since the 2011 malERA process, research has ranged from illuminating the basic biology of the development of sexual-stage parasites in humans and mosquitoes to evaluating operational approaches targeting infectious individuals in endemic communities. Additionally, a harmonised set of definitions relevant to malaria transmission and elimination has been developed (Box 1) [3]. However, there remains a need to further validate a ‘toolkit’ of metrics and associated surveillance activities to characterise the infectious reservoir and measure malaria transmission that can be applied programmatically to direct and evaluate interventions and to quantify progress towards malaria elimination. There are multiple factors that contribute to malaria epidemiology including ecology, vectors, parasites, human biology and behaviour, and economic and health-system factors (see Box 1), and these collectively make up a given ‘typology’ of malaria. The selection of appropriate surveillance activities and metrics from this toolkit will not only need to reflect variations in malaria ‘typology’ (Box 1) [3], but will need to be adapted as malaria transmission declines (Fig 1).

**Box 1. Terminology**

**Malaria typologies**

Malaria typology is the characterisation of malaria epidemiology according to ecology (climate and environment) and other determinants of transmission for the purpose of guiding malaria interventions. Relevant ecologies include (but are not limited to) savannah, lowland plains and valleys, highlands, desert and oasis, forest and jungle, coastal and marshland, and urban or peri-urban. The unique features of malaria transmission in each ecological area are also strongly driven by region-specific vectors and parasites (species, biology, behaviour, insecticide and antimalarial drug susceptibility), human biology and behaviour, and economic and health-system factors. These are discussed more comprehensively in [4] and [5].

This paper discusses progress in the measurement and understanding of malaria transmission, highlighting the different malaria typologies in which transmission occurs (Box 1). This differentiation between typologies is needed to determine where existing strategies and systems can sufficiently achieve malaria elimination versus those where additional approaches or tools are required.

## Research agenda for characterising the reservoir of infection

### Detecting malaria: Infection versus transmission

Malaria infection and transmission can be detected and measured with a variety of metrics (Tables 1 and 2). Their suitability and discriminatory power, however, can vary widely across settings and populations. To reliably confirm clinical malaria, a minimum diagnostic sensitivity of 200 parasites/μL blood is required [6]. Microscopy and some rapid diagnostic tests (RDTs) meet this threshold [6]. In the absence of fever, some individuals will have parasitaemia levels detectable by microscopy and RDTs. These asymptomatic infections are particularly common in areas of high transmission (i.e., above 25 clinical cases per week per 1,000 persons) [7], where high levels of human immunity allow older individuals to carry relatively large parasite burdens chronically [8]. Such individuals would be detected within mass screen and treat (MSAT) programmes using currently available diagnostics. However, through the use of molecular amplification methods, it is now clear that many individuals harbour low-density malaria infections beneath the limit of detection of both microscopy and RDTs [9]. Meta-analyses indicate that molecular methods detect up to twice as many *P*. *falciparum* infections as RDT or microscopy [10], and approximately 5 times as many *P*. *vivax* infections [11,12]. This gap in sensitivity may be more pronounced when compared against ultra-sensitive molecular methods [13]. Lack of sensitivity of diagnostic detection is more acute for *P*. *vivax* infections, which circulate at lower parasite densities hampering accurate estimates of true prevalence. There are also other unique challenges presented by *P*. *vivax* that make characterising its transmission reservoir problematic (Box 2) [14–18].

**Table 1:** Summary of currently available entomological malaria transmission metrics.

Metric	Definition [3]	Measure of transmission	Sampling method and resolution	Discriminatory power
Entomological inoculation rate (EIR)	Number of infective bites received per person in a given unit of time, in a human population	Transmission intensity	Human landing collection; light traps Resolution: Household or community level	Insensitive at low transmission Lack of standardised sampling design Collected by malaria control programmes
Sporozoite rate (SR)	Percentage of female *Anopheles* mosquitoes with sporozoites in the salivary glands	Risk of infection	Human landing catch; baited traps; gravid traps Resolution: Community level	Insensitive at low transmission
Human biting rate (HBR)	Average number of mosquito bites received by a host in a unit of time, specified according to host and mosquito species	Risk of exposure	Human landing collection Resolution: Person or community level	Allows determination of the primary vector
Vectorial capacity	Rate at which given vector population generates new infections caused by a currently infectious human case	Efficiency of transmission	Derived from human biting rate, parasite inoculation period, mosquito to human density and mosquito survival Resolution: Community level	Measures potential, not actual, rate of transmission—includes no parasitological information Sensitive to changes in mosquito survival and biting behaviour but may not translate to significant change in human incidence Can be useful when infection rates are low and mosquito sampling difficult

**Table 2:** Summary of currently available malaria transmission metrics in humans.

Metric	Definition [3]	Measure of transmission	Method	Discriminatory power
Annual blood examination rate (ABER)	The number of people receiving a parasitological test for malaria per unit population per year	Level of diagnostic monitoring activity	Microscopy or RDT	Dependent on health-system provision
Case, confirmed	Malaria case (or infection) in which the parasite has been detected in a diagnostic test	Current transmission or incidence if data collection is repeated or routine	Microscopy or RDT positive	Insensitive at low transmission; saturates at high transmission Underestimates due to system inadequacies and poor health-seeking behaviour
Case, fever	The occurrence of fever (current or recent) in a person	Current transmission or incidence if data collection is repeated or routine	Reported or observed fever	Overestimates malaria infection
Proportion of fevers parasitaemic (PFPf)*	Proportion of fever cases found to be positive for *Plasmodium*	Current transmission or incidence if data collection is repeated or routine	Microscopy; RDT; NAAT	Depends on diagnostic sensitivity Insensitive at low transmission
Slide positivity rate (SPR)	Proportion of blood smears found to be positive for *Plasmodium* among all blood smears examined	Current transmission or incidence if data collection is repeated or routine	Microscopy	Depends on ABER Insensitive at low transmission
RDT positivity rate (RDT-PR)	Proportion of positive results among all RDTs performed	Current transmission or incidence if data collection is repeated or routine	RDT	Depends on RDT sensitivity Insensitive at low transmission
Parasite rate (PR)	Proportion of the population found to carry asexual blood-stage parasites	Current transmission or incidence if data collection is repeated or routine	Microscopy; RDT; NAAT	Depends on diagnostic sensitivity Insensitive at low transmission
Gametocyte rate (GR)	Percentage of individuals in a defined population in whom sexual forms of malaria parasites have been detected	Potentially infectious human population	Microscopy; NAAT	Depends on diagnostic sensitivity Insensitive at low transmission

*No WHO definition is available for this term.

Abbreviations: ABER, annual blood examination rate; GR, gametocyte rate; NAAT, nucleic acid amplification test; PF*Pf*, proportion of fevers parasitaemic; PR, parasite rate; RDT, rapid diagnostic test; RDT-PR, RDT positivity rate; SPR, slide positivity rate.

**Box 2. *P*. *vivax* and *P*. *ovale***

*P*. *vivax* and *P*. *ovale* have a dormant liver stage, the hypnozoite, which is undetectable by currently available diagnostic methods. Periodic reactivation of hypnozoites results in repeated blood-stage infection (relapses) occurring weeks, or even years, following the initial infection. As control efforts reduce the incidence of *P*. *falciparum* cases, *P*. *vivax* cases can remain relatively stable and become a greater proportion of malaria cases overall [16]. *P*. *vivax* is refractory to traditional vector control methods: hypnozoites enable the parasite to evade conditions unfavourable to transmission and will survive in the host following schizonticidal anti-malarial therapy. Without new anti-hypnozoite drugs or vaccines that could be used safely across entire populations, the *P*. *vivax*/*ovale* transmission reservoir cannot be targeted, making elimination of these parasites challenging in any setting.

**Key advances**

**Relapses drive transmission**

- In children in Papua New Guinea, 4 of every 5 *P*. *vivax* infections and 3 of every 5 *P*. *ovale* infections were caused by relapses [14].
- Both primary and relapse *P*. *vivax* infections generate gametocytes, which typically appear before clinical symptoms, and promote onward ‘silent’ transmission of the parasite [15].
- Estimating transmission using the typical entomological measures is of limited relevance when clinical disease can emerge from an individual not recently infected by a mosquito bite.

**Research needs**

**Detection of hypnozoites to inform targeted drug or vaccination strategies**

- Access to existing anti-hypnozoite therapy needs to be expanded where possible in order to reduce the burden of disease and minimise the risk of human-to-mosquito transmission via relapse.
- However, several barriers to mass drug administration (MDA) for *P*. *vivax* exist. The 8-aminoquinolines primaquine and tafenoquine are the only known anti-hypnozoite drugs. Both drugs are contraindicated in pregnancy and individuals with glucose-6-phosphate dehydrogenase deficiency [17,18]. Even if rapid, accurate point-of-care tests were available to exclude these individuals from treatment, a significant proportion of the population (typically >10%) will remain untreated.
- Without being able to identify hypnozoites, MSAT is of no practical value in reducing *P*. *vivax* or *P*. *ovale* transmission [14].
- Compared to *P*. *falciparum*, *P*. *vivax* and *P*. *ovale* present as much lower parasite densities; therefore, determining the appropriate limit of detection for new diagnostics will be a major challenge.

**Improve understanding of parasite-vector bionomics**

Parasites can be transported undetected into areas where malaria has been eliminated, leading to outbreaks and the reestablishment of transmission where conditions are receptive. More effort needs to be directed at understanding specific parasite vector interactions to develop targeted vector control strategies for *P*. *vivax*/*ovale* to reduce the risk of mosquito-to-human transmission.

Diagnosis and treatment of clinical malaria is vital for disease control, particularly if this can be rapidly implemented to reduce the likelihood of gametocyte production. There is also a good public health rationale for identifying and treating ‘asymptomatic’ malaria detectable with microscopy or RDTs, as it is increasingly recognised that this is associated with ongoing morbidity (e.g., anaemia, increased susceptibility to bacterial infections, and cognitive function; reviewed in [8]). If the aim is malaria elimination, the contribution of low-density infections to transmission needs to be considered given that, where data are available, low-density infections represent a significant proportion of malaria infections and can be the majority in low-endemic areas [9,10,19,20].

While the countries that have achieved malaria elimination to date have done so largely without specific attempts to detect and treat low-density parasitaemia, these may not be representative of malaria typologies in higher-transmission settings. In many areas, the persistence of malaria can occur despite high coverage of vector control measures and the availability of effective treatment, suggesting that novel approaches are needed for both surveillance and interventions that will accelerate the elimination process [19,21]. Furthermore, studies have documented the failure of strategies to reduce clinical malaria incidence and transmission, such as MSAT, when the transmission reservoir is not adequately identified and targeted with the currently available field diagnostics [22].

It follows that the cost-effectiveness of existing or novel surveillance methods and interventions in reducing malaria transmission cannot be predicted or evaluated unless the relative contribution to transmission of (1) clinical/symptomatic malaria, (2) asymptomatic parasitaemia (detectable by microscopy or RDT), and (3) low-density parasitaemia (not detectable by microscopy or RDT) are estimated for a particular setting. With an increasingly diverse array of potential approaches for malaria elimination [18], but with limited human and financial resources [23], characterising the contribution of low-density parasitaemia to transmission will help to focus elimination efforts.

### Low-density parasitaemia and transmission

There are currently no field diagnostics with sufficient sensitivity to identify low-density submicroscopic parasitaemia, though various approaches are under evaluation for performance and scalability (discussed in the malERA Refresh ‘Tools’ paper) [18]. However, even if all infected individuals could be identified, there is a need to understand who is infectious to mosquitoes and for how long.

Understanding the contribution of low-density parasitaemia to the infectious reservoir for a given malaria typology is critical to determine the diagnostic sensitivity required. It will also affect how much effort a programme should commit to detecting and treating these infections and when and where this effort is best deployed. As noted above, the proportion of low-density parasitaemia increases as transmission declines [9,10,19,20,24]. Recent findings from Senegal also suggest that the efficiency of human-to-mosquito transmission increases with decreasing transmission intensity [25].

Currently, the only way to measure human infectiousness is by feeding colony-reared mosquitoes either on humans directly (direct feeding assay [DFA] [26,27]) or on infected human blood via a membrane (direct membrane feeding assay [DMFA] [28]). A number of studies have used these methods to estimate the contribution of low-density infections to malaria transmission [29–34]. For example, studies in Burkina Faso using DMFA found that 28.7% (25 out of 87) of infectious individuals were microscopy negative, causing 17.0% of mosquito infections [29]. Similarly, in Thailand, DFA studies found that 21% (13 out of 62) individuals submicroscopic for either *P*. *falciparum* or *P*. *vivax* were able to infect mosquitoes [34]. These preliminary studies suggest that surveillance systems could be modified in the future to detect submicroscopic infections and direct transmission reduction efforts. However, understanding the relationship between infectivity as measured in feeding assays and the infectivity in natural transmission settings to local mosquitoes is still a major research challenge. Furthermore, few empirical studies have quantified the proportion of the overall population that is both submicroscopic and infectious, particularly in low-transmission settings (i.e., less than 8 clinical cases per week per 1,000 persons) [7]. This is needed to determine when and where treating low-density parasitaemia is critical for interrupting transmission and the diagnostic sensitivity required to target them. Mathematical models suggest that conventional diagnostics can detect 55% of the infectious reservoir, but with a 100-fold increase in sensitivity of detection level, i.e., from 200 to 2 parasites/μL of blood, up to 95% of infectious individuals could be identified [35]. This level of diagnostic sensitivity could transform our understanding of the malaria transmission reservoir, allowing the development and delivery of better strategies to disrupt transmission toward malaria elimination.

### Detecting gametocytes

All malaria infections have the capacity to produce gametocytes. Therefore, in the context of community chemotherapy programmes, treating any individuals who test positive for asexual parasites is a realistic programme aim. However, research tools that measure gametocytaemia are essential to further our understanding of transmission biology and to define the populations and individuals that drive transmission. Some studies have suggested that transmission efficiency may increase as malaria prevalence falls due to higher gametocyte densities. As the development of new transmission-blocking drugs and vaccines advances, understanding the factors that drive this transmission efficiency will be needed to determine in which settings interventions can be successfully trialled and/or implemented [25]. Although gametocytes can be identified using microscopy, they often exist at low densities and may circulate only transiently in the blood. RDTs do not differentiate between gametocytes and asexual parasites. The limit of detection of microscopy is 8–16 gametocytes/μL of blood [30,31]. Predictably, molecular methods are more sensitive, with 0.3 mature females/μL of blood detected with Pfs25 reverse transcription qPCR (RT-qPCR) and 1.8 mature males/μL of blood with Pfs230p RT-qPCR [36]. As gametocyte densities are low, the increased sensitivity of molecular methods considerably increases gametocyte detection rates. For example, a recent study in Kenya found that Pfs25 RT-qPCR detected gametocytes in 44% of the population compared with only 2.6% detected by microscopy [37].

While there is an overall positive association between mosquito infection rates and gametocyte density, there is also evidence of infectiousness for individuals with very low gametocyte densities [27,38]. As the majority of malaria infections are submicroscopic, even if only a small proportion of these individuals are infectious, the contribution to the transmission reservoir is potentially significant enough to impact elimination programmes.

Where data are available, they suggest differences between high- and low-transmission settings in the gametocyte density needed for human infectivity to mosquitoes. In African populations, submicroscopic *P*. *falciparum* gametocytaemia is common, and studies in Kenya have found that the majority of infectious children (43 out of 62) had submicroscopic gametocytaemia [30,31]. In contrast, in Cambodia, *falciparum*-infected subjects with detectable gametocytes by microscopy were significantly more likely than gametocyte-negative individuals to infect mosquitoes, and those with microscopy-detectable gametocytaemia were the source of the majority of all mosquito infections [39].

### Heterogeneity in the transmission reservoir

While data demonstrate an advance in our understanding of malaria transmission, they are limited and suggest the infectious reservoir differs across malaria typologies [24]. Most studies investigating human infectiousness have been conducted in high-transmission settings. There is a particular need for data from low-transmission and near-elimination settings, where temporal, spatial, and demographic heterogeneity in transmission can often be more pronounced. Longitudinal data characterising the transmission reservoir are also needed. These would not only allow more accurate assessments of the contributions of the different density infections but could also inform the sequence of intervention delivery needed to reduce transmission. Similarly, these data would inform the necessary intervention changes to most effectively transition countries from high to low transmission and ultimately elimination [40]. A key consideration is to advise when malaria control measures should be reoriented following elimination without the risk of reintroduction, particularly in the context of declining human immunity to malaria and the potential for outbreaks.

As transmission declines and heterogeneity increases, programmes need to adjust in order to respond to increasingly rare clinical cases. The persistence of residual transmission requires more aggressive and/or novel strategies, and targeting these areas will be key to local elimination. Significant progress has been made in approaches to identify transmission foci using a number of field-based, geo-spatial, and modelling approaches [41–53]. However, even where hotspots of malaria transmission can be identified, attempts to target these foci may fail against a background of low-level but widespread transmission [54]. Local implementation and high-coverage control interventions linked to surveillance information will be needed to adequately clear the reservoir at all levels of transmission.

Surveillance systems at low-transmission settings will also need to be equipped to monitor emerging insecticide and drug resistance [55,56] that may threaten the success of existing interventions [56]. Longitudinal monitoring of resistance markers via sentinel surveillance sites could prove invaluable for tracking risk of rebound or reintroduction. However, there are currently no field-based diagnostic tests for drug resistance, and more detailed information may be needed on local drug-resistance patterns in asymptomatic/low-density infections, particularly related to any changed infectiousness to mosquitoes.

## Research agenda for measuring transmission

Improved and validated metrics of transmission would enable the optimal design of control programmes and surveillance systems needed for malaria elimination [23]. This would include the ability to better track progress, confirm cases and foci, and identify and contain reintroduction of transmission, should it occur. Validated transmission metrics are also the key outcome to be measured in field trials evaluating the effectiveness of transmission-blocking interventions [18] and can be used to improve mathematical models assessing potential intervention combinations [7].

**Fig 2:**
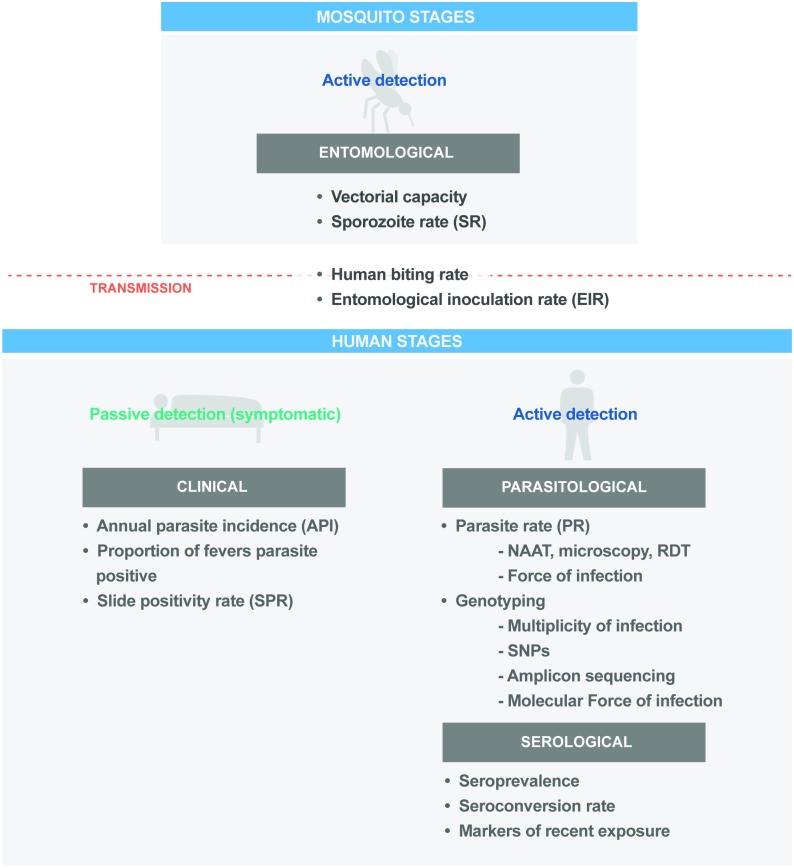
Key programmatic and research metrics across the malaria parasite transmission cycle. NAAT, nucleic acid amplification test; RDT, rapid diagnostic test.

Measures of malaria transmission can be defined at different points in the transmission cycle (Fig 2). Since 2011, progress has been made in understanding the advantages and limitations of transmission metrics across epidemiological settings [57,58]. Further work is needed to better quantify the correlations between metrics, standardise their application for use in programmatic surveillance activities, and develop and validate new metrics. However, it is necessary that transmission metrics are reliable and reproducible on a consistent basis and can be assembled through existing national systems.

### Entomological metrics

Between 30–40 species of *Anopheles* have been identified as vectors of human malaria, exhibiting varying feeding behaviours and preferences, habitats, and ecologies. Within this complexity, there is a need to standardise current metrics and develop more efficient sampling techniques [57] (Table 1). Whilst developments in sampling methods have been made to evaluate biting densities and infection rates [59–63], human landing collection (HLC) sampling remains the gold standard for providing epidemiologically relevant mosquito-to-human transmission metrics, despite inherent risks [64,65]. Alternative technologies to HLC are being tested that limit human exposure [66,67] and include traps with attractants that mimic a human host [68,69].

New approaches are particularly needed in settings where vector densities are low or heterogeneous. For example, reexamination of vectorial capacity using mathematical modelling to simulate settings with different baseline epidemiological and entomological characteristics has led to new insights into the effective deployment of vector control measures [70]. Technological advances in geolocation and mapping can precisely identify vector habitats that coincide with human activity and movement [71]. This information can be used to determine potential exposure points, enabling targeted sampling in these foci of transmission risk. Other innovative technologies include high-throughput technology, such as infrared spectrometry, to evaluate large samples of mosquitoes for vector age, species, and infection status [72–74], thus providing a measure of vector density and indicating the risk of malaria reintroduction. In this regard, as with parasite drug resistance, longitudinal monitoring of insecticide resistance via sentinel surveillance could prove invaluable.

### Human metrics

Current epidemiological metrics of malaria transmission in humans, diagnosed via passive and active systems, microscopy and RDTs, remain key for national malaria control programmes in tracking progress in the reduction of malaria cases and identifying outbreaks and epidemics (Table 2). These data are complemented with large-scale surveys, such as the Demographic and Health Surveys (DHS), the Malaria Indicator Surveys (MIS) and UNICEF Multiple Indicator Cluster Surveys (MICS). However, as transmission declines to low intensity, clinical cases become rare, slide and RDT positivity rates low, and transmission patterns increasingly heterogeneous.

To generate practical estimates of infection without excessive sampling, more sensitive diagnostics and/or combinations of diagnostic approaches are needed. While the utility of RDTs will need to be monitored in regions where deletions in the gene encoding HRP2 have been detected in the parasite population [75,76], research is currently underway to develop RDTs with detection thresholds corresponding to 10–20 parasites/μL or lower [77]. The development of highly sensitive nucleic acid–based tests for parasite detection [78,79], and hemozoin detection using nuclear magnetic resonance [80,81], is also ongoing and may be promising. While tests using molecular methods would increase the number of infections identified, their widespread deployment in low-transmission settings is probably not currently cost-effective for the identification of incident infections. Additionally, in recognition of heterogeneity, approaches should shift from tracking national or regional progress in malaria control towards targeted sampling and community-based surveys characterising transmission risk in key population groups. Once elimination has been achieved, maintaining ‘zero’ transmission will depend on the health system’s ability to identify any emergent malaria cases, triggering case-based investigation to determine the origin (local or imported) and prevent onward transmission.

### Metrics to understand transmission

Recent technical advances have produced a number of transmission metrics that are suitable for low-transmission settings (Table 3). Molecular force of infection (mFOI) and multiplicity of infection (MOI) both use parasite genotyping methods to assess the complexity of parasite infections [82]. mFOI can identify superinfected individuals that carry parasites from more than 1 infection, providing a more detailed measure of transmission compared to force of infection based on less sensitive methods (Table 2). Sequencing to determine parasite population structure can also be used to characterise transmission by measuring the genetic relatedness between infections in space and time. Other measures, such as allelic richness, can indicate the level of genetic diversity, which is expected to decline as transmission declines [83,84]. Even more refined sequencing approaches might be capable of assigning parasites as imported or local for monitoring the origin of infections.

**Table 3:** Advances in the development of metrics for measuring malaria transmission.

Metric	Definition	Measure of transmission	Method	Discriminatory power
Force of infection	Rate at which susceptible individuals contract malaria	Probability of transmission	Time from birth to first malaria episode; microscopic detection of parasites following successful antimalarial treatment	Difficult to measure Difficult to standardise Depends on diagnostic sensitivity Cannot differentiate superinfections
mFOI	The number of new parasite clones acquired by a host over time	Population-level transmission intensity Transmission heterogeneity	Cohort study >6 months with parasite genotyping	Highly sensitive for monitoring changes in malaria exposure Superinfections can be differentiated
MOI	The number of different parasite strains coinfecting a single host	Population-level transmission intensity Transmission heterogeneity	Parasite genotyping of positive samples	Saturates at high transmission Restricted by age dependency Insensitive at low transmission Highly sensitive to spatial heterogeneity Highly sensitive to increases in imported infection Less sensitive to changes in seasonality
Genotyping: SNPs or amplicon sequencing	Genetic diversity, i.e., number of alleles in a population Parasite signatures to map geographical relatedness of infection (i.e., spatial–temporal transmission)	Population-level transmission intensity Transmission heterogeneity Geographical tracking of transmission patterns	Haplotypes composed of >12 informative SNPs from single clone infections Haplotypic signatures from highly variable loci	Sensitive to changes in malaria exposure and spatial–temporal flow of infection Standardisation of measures needed Methods for analysis and interpretation of data needed
Antibody seroprevalence	The percentage of seropositive individuals in a population	Population-level transmission intensity	Seronegative or seropositive defined using appropriate cutoff points	Dependent on antibody target tested Saturates at high transmission Sensitive at low transmission
SCR	The rate (typically annual) by which seronegative individuals become seropositive upon malaria exposure	Population-level transmission intensity Temporal changes in transmission can be detected from a single sampling time point	Detection of antibodies in sera using serological assay (IFAT, ELISA, bead-based assays microarray)	Dependent on antibody target tested Restricted by age dependency Saturates at high transmission Sensitive at low transmission Sensitive to risk of malaria in absence of transmission

Abbreviations: ELISA, enzyme-linked immunosorbant assay; IFAT, Immunofluorescence Antibody Test; mFOI, molecular force of infection; MOI, multiplicity of infection; SCR, seroconversion rate.

Antibody seroprevalence and the seroconversion rate (SCR) exploit human antibody responses to characterise previous parasite exposure and are specific to a particular antigen or combination of antigens [85]. Studies using enzyme-linked immunosorbant assays (ELISAs) have shown serological measures correlate well with parasitological and entomological measures in describing transmission levels and spatial and demographic risk [86,87]. Uniquely, serology, when combined with age, allows retrospective examination of exposure history, including the effects of interventions and the absence of recent exposure in elimination settings. High-throughput platforms, such as microarray and bead-based multiplex assays, allow screening of large numbers of potential antigenic targets with specific characteristics [87,88–91]. Targets of interest include stage- or species-specific biomarkers, particularly for *P*. *vivax* [88], serological signatures of hypnozoite carriage [92], and vector-specific antigenic targets in mosquito saliva [93,94]. The programmatic applications of serology have yet to be fully tested, though various approaches are being evaluated, including serological markers of incident infections [89,95–109]. Research is currently underway to identify a variety of biomarkers indicative of recent infection that are detectable for different durations following parasite infection, allowing finer-scale estimation of time since infection.

For all these metrics, however, standardisation of methods is necessary, as well as a quantitative comparison to understand the relationship with existing and other new metrics. The development of operationally suitable platforms will ultimately be required to inform real-time or rapid response in programmatic settings. In relation to this, there needs to be a clearer understanding of what measures are needed to better define and monitor transmission, and what measures are useful for control programmes. New approaches to analyse metrics from different sources to improve estimates of transmission, or confirm its interruption, are needed. Looking to the veterinary world could be informative, where probability-based survey methods such as “freedom from infection” are used for animal disease surveillance in the food and agriculture industry [110]. These methods are based on defining the probability that a population is free of infection, allowing operational surveillance thresholds to be set based on the chosen sampling frame and the sensitivity of available diagnostics. Adapting these strategies for use in malaria surveillance will require tailoring the methods for specific malaria transmission measures.

### Multimetrics to characterise transmission in time and space

The increasing availability of spatial databases on parasite rate [111,112], serology, vectors [113], malaria genetic epidemiology [114], and human population movements [115–118], together with the increased flexibility and computational efficiency of mathematical and statistical modelling methods [119,120], have led to substantial advances in the spatial–temporal characterisation of malaria transmission intensity. To date, most of these methods have focused on a single metric of endemicity or have relied on parameters derived from small studies. However, dynamic models are being developed that will capture the effect of human population movements, and could incorporate multimetric ensembles to allow self-consistent mapping across the entire spectrum of transmission settings [7]. For these technologies to achieve the greatest impact, they will need to be linked to and used by control programmes to inform operational decision-making in real time.

## Summary

Considerable progress has been made not only in understanding the biology and epidemiology of malaria transmission but also in the development of new tools to more accurately quantify transmission; however, challenges remain and Box 3 summarises this Panel’s research and development agenda. The foremost of these is an incomplete understanding of the infectious reservoir in low-transmission and elimination settings, particularly the relative infectiousness of (1) asymptomatic individuals and (2) susceptible vector species across a variety of malaria typologies. The spatial and temporal heterogeneity at which these factors interact will change as countries transition to lower transmission intensity.

**Box 3. Research and development agenda**

**Characterising the reservoir**

Objective: Determine the relative contribution to transmission of symptomatic malaria, asymptomatic malaria detectable with microscopy or RDTs, and low-density infections detectable by molecular methods across different malaria typologies; data from low-transmission settings are particularly required.

**Research goals**

- Determine the kinetics of infectiousness of low-density parasitaemia.
- Determine the infectiousness of low-density gametocytaemia.
  - Refine mosquito feeding assays (DMFA or DFA) of human infectivity to mosquitoes and validate these against natural infectivity to local vector species.
- Determine the required sensitivity of field-based diagnostics to identify malaria infections contributing to transmission.
  - Continue to develop field-based molecular and serological diagnostics with sensitivities relevant for evaluation of infectious low-density parasitaemia and gametocytaemia.
  - Investigate non-invasive diagnostics of malaria infection and infectivity.
  - Develop hypnozoite diagnostics predictive of *P*. *vivax*/*P*. *ovale* relapse and subsequent infectivity.
  - Develop cost-effective programmatic triggers and protocols for the optimal deployment of transmission-based diagnostic tests and their incorporation within surveillance systems.
  - Evaluate the cost-effectiveness of programmatic actions and interventions directed by transmission-based diagnostics.
- Characterise changes in the transmission reservoir as transmission declines.
  - Conduct longitudinal studies in areas of declining transmission to investigate changes in the nature and distribution of the transmission reservoir.
  - Evaluate which surveillance activities and metrics are most informative and cost-effective for programmatic goals.
  - Develop operational methods to rapidly identify antimalarial drug-resistant parasites and insecticide-resistant vectors.
- Determine the relevance of spatial–temporal heterogeneity in the transmission reservoir to the acceleration of elimination.
  - Identify foci of residual transmission.
  - Identify areas at risk for outbreaks and the reestablishment of malaria transmission following local elimination.

**Measuring transmission**

Objective: To develop a standardised and validated ‘toolkit’ of metrics and surveillance activities for characterising the infectious reservoir and measuring malaria transmission, which can be applied programmatically to direct interventions, evaluate interventions, and quantify progress towards malaria elimination.

**Research goals**

- Development of entomological as well as human measures and surveillance of transmission.
  - Continue to develop alternatives to HLC sampling for entomological measures of transmission risk.
- Continued quantification of the relationships between different metrics of transmission.
- Develop validated metrics for use in low-transmission settings and in the absence of transmission.
  - Continue to develop methods for evaluating transmission risk in low-transmission settings or in the absence of transmission.
  - Evaluate multimetric combinations for the efficient integration and analysis of low-intensity and/or heterogeneous transmission.
  - Evaluate the most cost-effective and informative metrics aligned to programmatic goals as transmission declines.
- Develop validated metrics for the evaluation of new tools directed at transmission interruption.

The absolute and relative incidence of clinical and asymptomatic infections can vary widely between different low-transmission settings. Transmission can occur as focal outbreaks caused by human and vector migration. It can also persist for long periods despite aggressive control strategies or quickly rebound after reaching zero. These scenarios are caused by varying patterns of malaria risk across demographic groups, vectors, and parasite species in different ecological settings, which may not be easily captured by simple incidence and prevalence measures.

The application of new and/or refined metrics for routine surveillance activities or research-specific contexts requires investigation. This needs to be done in the context of existing standard measures and the newer data collection platforms to understand the true utility. Metrics will also need to be optimised for the quality of the healthcare system in which they will be implemented. The same applies to the infectious reservoir. Whilst its characterisation across different transmission settings is important, translating this information into actionable programmatic decisions will be key to achieving zero malaria transmission.

## Abbreviations

ABER: annual blood examination rate
DFA: direct feeding assay
DHS: Demographic and Health Surveys
DMFA: direct membrane feeding assay
ELISA: enzyme-linked immunosorbant assay
HLC: human landing collection
malERA: Malaria Eradication Research Agenda
MDA: mass drug administration
mFOI: molecular force of infection
MICS: Multiple Indicator Cluster Surveys
MIS: Malaria Indicator Surveys
MOI: multiplicity of infection
MSAT: mass screen and treat
NAAT: nucleic acid amplification test
RDT: rapid diagnostic test
RT-qPCR: reverse transcription qPCR
SCR: seroconversion rate
